# The Classification of Myeloproliferative Neoplasms: Rationale, Historical Background and Future Perspectives with Focus on Unclassifiable Cases

**DOI:** 10.3390/cancers13225666

**Published:** 2021-11-12

**Authors:** Marco Pizzi, Giorgio Alberto Croci, Marco Ruggeri, Silvia Tabano, Angelo Paolo Dei Tos, Elena Sabattini, Umberto Gianelli

**Affiliations:** 1 Surgical Pathology and Cytopathology Unit, Department of Medicine—DIMED, University of Padua, 35128 Padua, Italy; angelo.deitos@unipd.it; 2 Department of Pathophysiology and Transplantation, University of Milan, 20122 Milan, Italy; giorgio.croci@unimi.it (G.A.C.); umberto.gianelli@unimi.it (U.G.); 3 Division of Pathology, Fondazione IRCCS Ca’ Granda, Ospedale Maggiore Policlinico, 20122 Milan, Italy; 4 Department of Hematology, San Bortolo Hospital, 36100 Vicenza, Italy; marco.ruggeri@aulss8.veneto.it; 5 Laboratory of Medical Genetics, Foundation IRCCS Ca’ Granda, Ospedale Maggiore Policlinico, 20122 Milan, Italy; silvia.tabano@unimi.it; 6 Haematopathology Unit, IRCCS Azienda Ospedaliero-Universitaria di Bologna, 40138 Bologna, Italy; elena.sabattini@aosp.bo.it

**Keywords:** Myeloproliferative neoplasms, WHO Classification, MPN-U, myeloid disorders

## Abstract

**Simple Summary:**

Myeloproliferative neoplasms (MPNs) are clonal hematological disorders, characterized by increased proliferation of the myeloid lineages in the bone marrow. Since their original recognition by William Damashek in 1951, MPNs have been extensively investigated at a clinical-pathological and molecular level. This prompted a progressive refinement of their classification and diagnostic criteria. Uncertainties nonetheless remain in a small (yet consistent) subset of cases, characterized by unconventional and/or overlapping clinical-pathological features. Such cases (referred to as MPN, unclassifiable [MPN-U]) encompass a broad spectrum of entities, including early phase MPNs, terminal (i.e., fibrotic) MPNs, MPNs associated with inflammatory or neoplastic disorders, and poorly characterized MPNs with clinical-pathological mismatch or atypical molecular features. In this review, we discuss the rationale behind the classification and diagnostic criteria of MPNs, focusing on the still open issues concerning MPN-U.

**Abstract:**

Myeloproliferative neoplasms (MPNs) are a heterogeneous group of clonal hematopoietic stem cell disorders, characterized by increased proliferation of one or more myeloid lineages in the bone marrow. The classification and diagnostic criteria of MPNs have undergone relevant changes over the years, reflecting the increased awareness on these conditions and a better understanding of their biological and clinical-pathological features. The current World Health Organization (WHO) Classification acknowledges four main sub-groups of MPNs: (i) Chronic Myeloid Leukemia; (ii) classical Philadelphia-negative MPNs (Polycythemia Vera; Essential Thrombocythemia; Primary Myelofibrosis); (iii) non-classical Philadelphia-negative MPNs (Chronic Neutrophilic Leukemia; Chronic Eosinophilic Leukemia); and (iv) MPNs, unclassifiable (MPN-U). The latter are currently defined as MPNs with clinical-pathological findings not fulfilling the diagnostic criteria for any other entity. The MPN-U spectrum traditionally encompasses early phase MPNs, terminal (i.e., advanced fibrotic) MPNs, and cases associated with inflammatory or neoplastic disorders that obscure the clinical-histological picture. Several lines of evidence and clinical practice suggest the existence of additional myeloid neoplasms that may expand the spectrum of MPN-U. To gain insight into such disorders, this review addresses the history of MPN classification, the evolution of their diagnostic criteria and the complex clinical-pathological and biological features of MPN-U.

## 1. Introduction

The Myeloproliferative Neoplasms (MPNs) are a heterogeneous group of clonal hematopoietic stem cell disorders, characterized by increased proliferation of the myeloid lineages in the bone marrow (BM) [1]. These disorders primarily affect adult to elderly patients with a cumulative annual incidence of 1–5 cases/100,000 [2]. Males are more commonly affected than females, yet relevant differences exist in the epidemiology of each entity. The clinical-laboratory features of MPNs depend on the main lineage of differentiation, the molecular landscape, and the disease stage. The life expectancy of untreated patients is reduced compared to the general population, given the high risk of thrombotic and hemorrhagic complications, BM failure, end-organ damage and blast transformation upon disease progression [3]. The MPNs are traditionally classified into four sub-groups, which include (i) Chronic Myeloid Leukemia (CML); (ii) classical Philadelphia-negative MPNs (Polycythemia Vera, PV; Essential Thrombocythemia, ET; Primary Myelofibrosis, PMF); (iii) non-classical Philadelphia-negative MPNs (Chronic Neutrophilic Leukemia, CNL; Chronic Eosinophilic Leukemia, CEL); and (iv) MPN, unclassifiable (MPN-U) [1]. This classification stems from the decades-long cooperation of hematologists, pathologists, and molecular biologists, which aimed at a better understanding of the pathophysiology, diagnostic criteria, and management of such disorders.

Far from being solely academically relevant, the rationale of MPN classification is instrumental to address the open issues on these tumors. It also supports everyday clinical practice, providing theoretical and practical clues for the diagnosis of difficult cases. To this aim, the present review will address the history of MPN classification and the rationale behind its changes over the years. Special attention will be paid to classical Philadelphia-negative MPNs and MPN-U, which are the most heterogeneous and challenging of all MPNs.

## 2. History and Rationale of MPN Classification

The history of MPNs dates back to the nineteenth century, when reports of extreme leukocytosis and/or erythrocytosis highlighted a class of hematological disorders, characterized by markedly increased hematopoiesis [4]. In 1951, William Damashek proposed the first classification of such conditions, which he originally named *myeloproliferative disorders* (MPDs). Based on Damashek’s proposal, the MPDs included CML, PV, ET (referred to as *megakaryocytic leukemia*), PMF (*idiopathic* or *agnogenic myeloid metaplasia*) and erythroleukemia (Di Guglielmo syndrome) [5]. This classification was based mainly on clinical and laboratory findings, with limited contribution of BM morphology (Table 1; panel A).

Over the years, clinical-pathological studies highlighted consistent differences in the biology and prognosis of MPDs and suggested splitting these entities in two sub-groups: (i) *myeloleukemia syndromes* (CML and erythroleukemia), characterized by single lineage hematopoiesis and a high tendency of blast transformation; and (ii) *myeloproliferative syndromes* (PV, ET, and PMF) with a more indolent clinical course, non-destructive hematopoiesis and higher rates of fibrotic evolution [6]. While based on disputable assumptions, this distinction was essentially correct, as Di Guglielmo syndrome was to be later listed among acute leukemias/myelodysplastic syndromes [7] and CML disclosed molecular and clinical features clearly distinct from all other MPNs [1,8].

Damashek’s classification was embraced in 1976 by the WHO Classification of hematological tumors with the notable inclusion of *chronic myeloproliferative diseases (CMPDs), unclassified* as a further diagnostic category [9] (Table 1; panel A). According to the WHO standards, such a diagnosis had to be rendered in Philadelphia-negative cases lacking clear-cut features of any other definitive entity.

In the 1980s and early 1990s, BM histology gained progressive relevance in the study of CMPDs and prompted histology-based classifications, including the Working Classification of CMPDs [10] and the so-called Hannover Classification [11]. The former identified three groups of entities: (i) *typical CMPDs* (corresponding to Damashek’s classical PV, ET and CML); (ii) *intermediate* or *transitional CMPDs* (characterized by unconventional clinical and/or pathological features); and (iii) *transformed CMPDs*, representing advanced (fibrotic or blast phase) disorders (Table 1; panel B). While addressing the complex histologic landscape of CMPDs, the Working Classification was hampered by the high rate (23%) of unclassifiable cases. The Hannover Classification partially overcame these limitations by adopting a simplified classification scheme and introducing a new clinical-pathological entity (*chronic megakaryocytic-granulocytic myelosis*) that corresponds to early phase PMF [12] (Table 1; panel B). Nonetheless, uncertainties remained regarding CNL and CEL (still considered within the spectrum of CML) and on the actual existence of primary (i.e., idiopathic) cases of Myelofibrosis (MF). These issues were addressed by clinical and molecular studies on chronic leukemias [13,14] and by seminal works on PMF from the Cologne group [12]. The 2001 WHO Classification of hematopoietic tumors endorsed these studies and provided an expanded list of CMPDs, including CEL, CNL and Chronic Idiopathic (i.e., Primary) MF as separate entities [15] (Table 1; panel C). This classification relied on the integration of clinical, histological and genetic data, much in the way of the REAL Classification for lymphoid tumors that had been proposed a few years before [15,16]. While not universally accepted [17], this clinical-pathological approach marked a turning point in the history of MPNs.

Following the 2001 WHO Classification, the molecular bases of myeloid tumors were deeply investigated with the discovery of *JAK2* and *MPL* mutations in Philadelphia-negative CMPDs [18], *KIT* mutations in Mastocytosis [19], and *PDGFRA*, *PDGFRB,* and *FGFR1* rearrangements in subsets of myeloid/lymphoid neoplasms with eosinophilia [20]. All of this was included in the 2008 WHO Classification (Table 1; panel C), which definitively recognized the neoplastic nature of such conditions and abandoned the term CMPD in favor of MPN. Mastocytoses were also listed within the spectrum of MPNs, while myeloid/lymphoid neoplasms with *PDGFRA*, *PDGFRB,* and *FGFR1* rearrangements were considered separately [21,22]. The 2016 revision of the WHO Classification maintained this scheme with the notable exception of Mastocytoses, which were moved to a separate section due their unique clinical-pathological features [1,23] (Table 1; panel C). In keeping with prior approaches, the 2001, 2008, and 2016 WHO Classifications retained a distinct diagnostic category for MPN-U.

**Table 1:** Evolution of the Classification of MPNs.

**A. EARLY CLINICALLY-ORIENTED CLASSIFICATIONS**		
**Damashek’s Classification** [5]	**1976 WHO Classification** [9]	
Chronic Myeloid Leukemia Polycythemia Vera Idiopathic or Agnogenic Myeloid Metaplasia of Spleen Megakaryocytic Leukemia Erythroleukemia	Chronic Myeloid Leukemia Chronic Myeloid Leukemia variants - neutrophilic leukemia - eosinophilic leukemia - basophilic leukemia Chronic erythremia Polycythemia Vera Idiopathic Thrombocythemia Myelosclerosis with Myeloid Metaplasia CMPD, unclassified	
**B. HISTOLOGICALLY-ORIENTED CLASSIFICATIONS**		
**Working Classification** [10]	**Hannover Classification** [11]	
***a. Typical CMPDs*** Erythrocytic myelosis Granulocytic myelosis Megakaryocytic myelosis ***b. Intermediate CMPDs*** Erythrocytic myelosis. atypical Megakayocytic-granulocytic myelosis Megakaryocytic myelosis, immature/pleomorphic ***c. Transformed CMPDs*** Myelofibrosis/Osteomyelosclerosis Myelofibrosis/Osteomyelosclerosis with Blast Crisis Blast Crisis	***a. Primary diseases*** Chronic Myeloid Leukemia - common (granulocytic) type - megakaryocytic type - overlapping type - other types (chronic neutrophilic, eosinophilic or basophilic leukemia; juvenile Chronic Myeloid Leukemia) Polycythemia Vera Primary (Idiopathic) Thrombocythemia Chronic megakaryocytic-granulocytic myelosis ***b. Advanced disease*** Excess of Blasts and Blast Crisis Myelosclerosis and Myelofibrosis	
**C. INTEGRATED CLINICAL-PATHOLOGICAL CLASSIFICATIONS**		
**2001 WHO Classification** [15]	**2008 WHO Classification** [20]	**2016 WHO Classification** [1]
Chronic Myelogenous Leukemia Chronic Neutrophilic Leukemia Polycythemia Vera Chronic Idiopathic Myelofibrosis Essential Thromobocythemia Chronic Eosinophlic Leukemia CMPD, unclassifiable	Chronic Myelogenous Leukemia, *BCR-ABL1* positive Chronic Neutrophilic Leukemia Polycythemia Vera Primary Myelofibrosis Essential Thrombocythemia Chronic Eosinophlic Leukemia, NOS Mastocytosis MPN, unclassifiable	Chronic Myeloid Leukemia, *BCR-ABL1* positive Chronic Neutrophilic Leukemia Polycythemia Vera Primary Myelofibrosis (PMF) - prefibrotic/early PMF - overt PMF Essential Thrombocythemia Chronic Eosinophlic Leukemia, NOS MPN, unclassifiable

## 3. Evolution of the Diagnostic Criteria for MPNs

In the early 1970s, the Polycythemia Vera Study Group (PVSG) proposed the first diagnostic criteria for MPNs [24,25]. Being designed for clinical trials, the PVSG consensus had high specificity, but limited sensitivity [4,26]. This prompted the elaboration of more sensitive, integrated clinical-pathological criteria, including the Rotterdam [26] and the 2001 WHO criteria for MPNs [15]. The 2008 and 2016 WHO Classifications endorsed this approach, also considering the molecular and pathogenic features of each entity [1,21].

### 3.1. Evolution of the Diagnostic Criteria for PV

The PVSG diagnostic criteria for PV relied primarily on clinical and laboratory findings and adopted red cell mass (RCM) as the sole indicator of erythroid load. BM evaluation had little (if any) role in the diagnostic workup [4,24]. While still backed by some authors [17], the PVSG criteria were hampered by (i) limited sensitivity; (ii) the need for nuclear medicine to estimate RCM; and (iii) the poor reproducibility of RCM assessment. This was addressed by the Rotterdam [26] and 2001 WHO criteria, which added hemoglobin (Hb) as an indicator of erythroid mass and included BM histology to improve diagnostic accuracy (Table 2) [15]. The discovery of *JAK2* mutations prompted major changes in the diagnosis of PV, as it improved the distinction from reactive erythrocytosis and allowed the detection of early stage disease. Moving from these assumptions, the 2008 WHO Classification listed *JAK2* mutations among the major diagnostic criteria for PV. It also lowered Hb levels and added hematocrit as a further surrogate of RCM. As *JAK2* mutations are not unique to PV, minor (histological and laboratory) criteria were listed to increase diagnostic specificity [22]. Despite these changes, the 2008 WHO criteria still missed subsets of early phase PV, characterized by overt thrombocytosis and mild erythrocytosis [27]. To detect these “masked PV” cases, the 2016 WHO Classification further lowered the diagnostic thresholds of Hb and hematocrit. The decrease in specificity was compensated with the promotion of BM histology to a major diagnostic criterion (Table 2) [28].

### 3.2. Evolution of the Diagnostic Criteria for ET

Consensus diagnostic criteria for ET were proposed by the PVSG in 1986 [25]. They were designed mainly to exclude alternative causes of thrombocytosis and gave little relevance to BM evaluation (Table 3). Over time, compelling evidence demonstrated the heterogeneity of MPNs with thrombocytosis and highlighted the existence of PV and early PMF cases clinically mimicking ET. The PVSG criteria inadequately distinguished such entities, with detrimental consequences for patient management [12,29,30,31]. All of this prompted the development of new diagnostic standards (the Rotterdam and 2001 WHO criteria), based on the integration of clinical and pathological data [15]. 

The 2008 WHO Classification maintained this approach and integrated clinical-pathological and molecular data to increase diagnostic sensitivity, lower platelet thresholds and simplify exclusion criteria [21]. The 2016 WHO Classification made minimal changes to this diagnostic workup, just adding *CALR* mutations to the molecular derangements typical of ET (Table 3) [1].

### 3.3. Evolution of the Diagnostic Criteria for PMF

The diagnostic criteria for PMF have undergone relevant changes over the years. Prior to the Cologne consensus of 1996, a diagnosis of MF was made only upon documentation of (i) severe BM fibrosis; (ii) anemia; (iii) poikilocytosis; (iv) leukoerythroblastosis; and (v) massive splenomegaly [12]. The description of early phase PMF prompted broader diagnostic standards, considering both pre-fibrotic and full-blown cases. This novel approach was first adopted by the Cologne criteria for Idiopathic MF, which have inspired all subsequent developments [32]. The 2001 WHO Classification endorsed the distinction between pre-fibrotic and overt PMF, but did not provide clear-cut diagnostic criteria for such entities [15]. These limits were addressed by the 2008 WHO Classification, which proposed major and minor criteria based on clinical-pathological and molecular parameters. Early phase and overt disease, however, were lumped together and phase-specific diagnostic parameters were lacking [21]. Separate criteria were eventually proposed by the 2016 WHO Classification, which also added *CALR* mutations to the list of PMF-associated molecular changes (Table 4) [1]. Of note, the 2008 and 2016 WHO Classifications adopted distinct diagnostic criteria for secondary forms of MF (i.e., post-ET and post-PV MF). 

## 4. Myeloproliferative Neoplasms, Unclassifiable (MPN-U)

Approximately 5–10% of all MPNs display some clinical, morphological, or molecular features of MPN, but do not fulfill the aforementioned diagnostic criteria or present ambiguous features. These cases (defined as MPN-U) are traditionally clustered into three clinical-pathological groups: (i) early phase MPNs; (ii) advanced fibrotic phase MPNs; and (iii) MPNs with concurrent inflammatory or neoplastic disorders obscuring the clinical-histological picture [1,33].

In early phase MPN-U, clinical and/or morphological features of a specific entity are not fully developed. With widespread application of molecular screening, pathologists are increasingly confronted with such cases, which account for the vast majority of MPN-Us. Moreover, roughly 50% of MPNs with splanchnic vein thrombosis (SVT) have a clinical phenotype not diagnostic of a specific entity. Until definite criteria are met, such cases are better diagnosed as MPN-U. In some instances, the first diagnosis of MPN is performed in the fibrotic phase and stromal alterations (collagen deposition, increased micro-vessel density, sinusoids ectasia, and bone remodeling) are so advanced that the differential diagnosis between PMF and secondary MF is not feasible [33].

The clinical features of MPN-U at presentation are variable: increased blood cell counts (thrombocytosis, leukocytosis and/or polyglobulia) without organomegaly are more frequent in early phase MPN-U, while cytopenia (thrombocytopenia, leukopenia, and anemia) with splenomegaly and hepatomegaly are usually associated with advanced disease stages. The diagnosis can be challenging and requires the exclusion of mimicking conditions, such as infections and toxin or drug exposure (growth factors, cytokines, or immunosuppressive drugs). In this context, identifying mutations in MPN driver genes (*JAK2*, *CALR*, or *MPL*) or other myeloid neoplasm-associated genes (*ASXL1*, *EZH2*, *TET2*, *IDH1*, *IDH2, SRSF2*, and *SF3B1*) hints to the clonality of hematopoiesis and allows distinction from reactive conditions [1,34]. The same holds true for cytogenetic abnormalities, which can be found in approximately 30% of cases. It is worth noting that a diagnosis of MPN-U cannot be made in cases with genetic lesions defining other myeloid neoplasms (*BCR-ABL1* fusion, rearrangements of *PDGFRA*, *PDGFRB*, *FGFR1* and/or *PCM1-JAK2* fusion), if clinical data are incomplete or if biopsy samples are inadequate [1,33].

The evolution and prognosis of MPN-U is variable. In some cases, follow-up allows re-classification within a specific MPN and the prognosis is that of such disease. Patients with MPN-U in the early phases tend to have a relatively favorable prognosis, akin to that of ET or early phase PMF. In these cases, thrombotic-hemorrhagic events are the most frequent complications. In advanced and fibrotic stages, the prognosis is instead poor with possible progression to accelerated or blast phase [33,35].

Literature studies and our own data confirm the heterogeneity of the MPN-U spectrum. As previously highlighted, cases presenting with SVT constitute a unique subtype of MPN with misleading morphology and clinical features. In a study on 29 patients of this type, we found 11/29 (37.9%) cases morphologically consistent with PV, 11/29 (37.9%) cases reminiscent of PMF, and 6/29 (20.1%) of ET. Molecular analyses identified the *JAK2^V617F^* mutation in 27/29 (93.1%) patients and *MPL^W515K^* mutation in 1/29 (3.5%) cases. The remaining case (1/29 [3.5%]) lacked *JAK2*, *CALR,* and *MPL* mutations and was considered “triple-negative” (TN). According to the 2008 WHO Classification criteria, 3/29 (10.3%) patients were diagnosed with PV, 11/29 (37.9%) with PMF, and 2/29 (7.0%) with ET. The remaining 13/29 (44.8%) cases fell into the MPN-U category due to discrepancies in morphological and clinical features [36]. In another large cooperative study, we assessed the clinical-pathological features of 71 MPN-Us, in which diagnosis was based on the 2008 WHO Classification criteria. Morphologically, 26/71 (36.6%) cases showed ET-like, 26/71 (36.6%) PMF-like, and 15/71 (21.1%) PV-like morphology. Interestingly, 42/71 (59.2%) cases disclosed severe (MF-2 or MF-3) BM fibrosis. Clinically, Hb levels and white blood cell (WBC) counts were frequently normal, while the median platelet count and lactate dehydrogenase (LDH) levels of the whole series were increased. Splenomegaly was documented in 31/71 (43.7%) patients. On the molecular level, the *JAK2^V617F^* mutation was detected in 51/71 (71.8%), *CALR* mutations in 8/71 (11.2%), and the *MPL^W515L^* mutation in 2/71 (2.8%) patients. Two out of 71 (2.8%) cases resulted TN, while 8/71 (11.3%) patients had only partial molecular characterization (i.e., *JAK2* negativity with no data on *CALR* and *MPL* status) [33]. The reclassification of these cases according to the revised 2016 WHO criteria yielded relevant changes: among the prodromal/early phase MPN-Us, 21/29 (72.4%) cases were re-classified as ET (20/21 [95.2%]) or PV (1/21 [4.8%]). Of the remaining 8/29 (27.6%) cases, 3/29 (10.3%) displayed morphologic features of ET but lacked the required platelet threshold, 3/29 (10.3%) were consistent with pre-PMF but failed to meet the clinical criteria for such diagnosis, and 2/29 (7.0%) showed PV-like morphology without definite clinical correlates. In the advanced fibrotic group, 31/42 (73.8%) cases were re-classified as advanced PMF (25/42 [73.8%]) and PV (6/42 [14.3%]). Of the remaining 11/42 (26.2%) cases, 7/42 (16.7%) displayed PMF-like morphology but lacked any of the minor diagnostic criteria, while 4/42 (9.5%) were morphologically consistent with PV, but failed to meet the required Hb threshold values (3 cases) or lacked *JAK2* mutations [35]. Overall, these results indicate that refining the diagnostic criteria for conventional MPNs significantly reduces the rate on unclassifiable cases. A consistent proportion of MPNs nonetheless escapes any attempt of classification and may represent a persistence of poorly characterized subsets of myeloid neoplasms. These conclusions are in keeping with a recent study from Deschamps et al. [37], which described the clinical, pathological, and molecular features of a series of 82 MPN-Us. In this cohort, the median age at diagnosis was 49 years (range: 13–79), with a slight female predominance (46/82 [56.1%]). Thrombocytosis was present in 64/82 (78.0%) patients with a median platelet count of 650 × 10^9^/L. Hb levels and WBC counts were within the normal range. Clinical findings included splenomegaly (22/82 [26.8%]), pruritus (30/82 [36.6%]), constitutional symptoms (24/82 [29.3%]), and transfusion dependency (2/82 [2.4%]). The peripheral blood film displayed leukoerythroblastic features (5/71 [7.0%]), ‘tear drop’ poikilocytes (13/71 [18.3%]), and large granular lymphocytes (14/71 [19.7%]). Most cases (62/82 [75.6%]) carried mutations in MPN driver genes, mainly involving *JAK2* (46/82 [56.1%]), while 20/82 (24.4%) cases were TN; a subset of patients underwent further investigation, which allowed the identification of variants affecting *TET2*, *ASXL1*, *SRSF2*, and *RUNX1*. Interestingly, reticulin fibrosis was mild (MF-1) to absent (MF-0) in the majority of cases. In keeping with the updated 2016 WHO diagnostic criteria, this suggests that most new MPN-Us are diagnosed in the early, pre-fibrotic stage.

## 5. Challenges and Perspectives in the Classification of MPN-U

**Figure 1:**
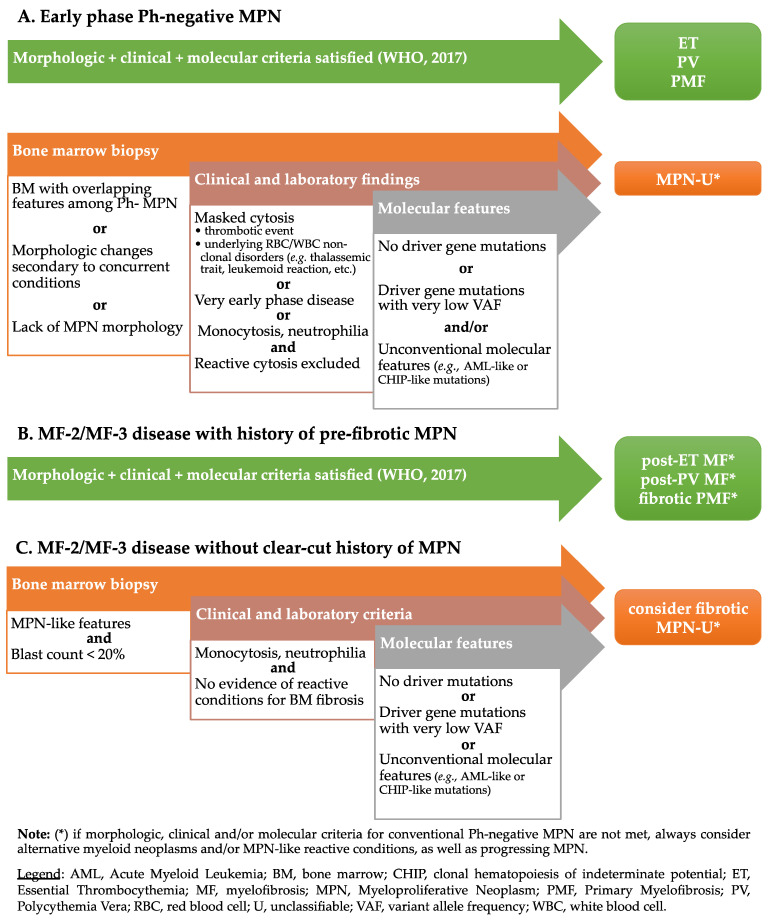
Diagnostic flow chart for patients with suspected MPN.

When dealing with a patient with suspected Philadelphia-negative MPN, several scenarios can be recognized (Figure 1). In many instances, the clinical, histological, and molecular findings allow a definite diagnosis [38,39]. In rare cases, however, clinical-pathological and/or molecular mismatch contrasts with WHO-defined entities and prevents definite conclusions. Such cases may expand the spectrum of acknowledged MPN-Us, pinpointing to future developments on MPN classification. The following paragraphs will address these unconventional cases, presenting real-life examples and discussing a general framework for their interpretation.

### 5.1. MPN with Clinical-Morphological Mismatch

In the proper clinical setting, histology provides invaluable information to make a definite diagnosis of MPN. In particular, megakaryocyte morphology and hypercellular hematopoiesis represent cardinal diagnostic traits, the latter being minor-to-absent in ET and in advanced phases of fibrotic MPN. Particularly in early phase MPNs, the lack of fully-developed morphologic features may conceal a diagnosis of clonal disease, or its punctual classification. Furthermore, morphologic anomalies of the hematopoietic lineages and/or increased monocyte, eosinophil, and basophil counts may challenge a diagnosis of MPN, leaning towards myelodysplastic syndrome (MDS) or MDS/MPN conditions.

#### 5.1.1. Cases with MPN-like Clinical Findings, Lacking MPN Morphologic Criteria

As previously reported, morphologic patterns on BM biopsy constitute a major and necessary criterion to make a diagnosis of MPN (Table 2, Table 3 and Table 4), despite that histologic evaluation is hampered by some degree of interobserver variability. To this regard, the differential diagnosis between early PMF and ET constitutes critical and long-debated grounds for interpretation [40]. In this setting, a provisional diagnosis of MPN can be provided with the caveat that definite subtyping may require follow up. In rarer instances (which are exemplified hereafter) unconventional clinic-pathologic features may hinder not only the definition of a specific MPN, but also the diagnosis of MPN itself.

##### Case 1—Synopsis and Discussion

**Figure 2:**
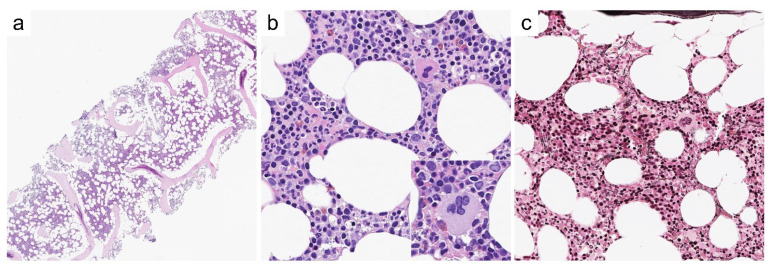
Representative histological features of case #1. BM biopsy shows cellularity within normal limits (**a**; H/E, 20×) with accumulation of hematopoiesis in central marrow spaces and marginalization of the adipose tissue close to the bony trabeculae. Maturation of erythroid and myeloid lineages is preserved (**b**; H/E, 200×), but scattered, large megakaryocytes are noted (**b**, *inset*). Reticulin network is unremarkable (**c**, Gomori silver stain, 200×).

A 41-year old male presents with a 5-year history of erythrocytosis and high hematocrit levels. Laboratory test at presentation disclose high Hb (17.8 g/dL) and Hct (54%), normal WBC (6.7 × 10^9^/L) and platelet counts (203 × 10^9^/L), and erythropoietin (Epo) levels below local laboratory ranges (3.9 mU/mL [4.3–29]). A *JAK2^V617F^* mutation with 2.8% variant allele frequency (VAF) is detected on peripheral blood granulocytes (data on exon 12 and/or additional exon 14 mutations not available). The overall clinical presentation is suspected for PV and prompts a BM evaluation. A BM biopsy (Figure 2) features cellularity within age-related limits, preserved maturation of erythroid and myeloid lineages, and only a minor increase in megakaryocytes. The latter are sparse in distribution, with predominant mature-type morphology and only rare, scattered large forms. Thus, the lack of clear-cut major criterion 2 (Table 2) does not allow a diagnosis of PV and warrants a provisional characterization as MPN-U.

In the present case, the histology is not inconsistent with the clinical diagnosis of PV, but it is not robust (particularly, some clustering of megakaryocytes with scattered large forms are disputable). A pre-morphologic diagnosis of MPN (i.e., a diagnosis made without full-blown MPN histology) is not common [41]. Indeed, it may occur in clinical settings where awareness of early phase MPNs suggests comprehensive laboratory and molecular screening, including quantitative PCR for low-burden driver gene mutations [42]. In such a context, it is worth noting that low VAF of *JAK2^V617F^* may be due to multiple base mutations in exon 14, which may hamper primer annealing and affect the quantification of the mutational burden [41]. Thus, searching for additional exon 14 mutations may be justified in cases with inexplicably low burdens of *JAK2^V617F^*.

#### 5.1.2. Cases with MPN-like Morphology, Lacking MPN Clinical Criteria

Far more common than the previous case is the instance of histopathologic and/or molecular findings fully supportive of MPN, but with clinical features obscuring either the diagnosis or the subtyping of the clonal process.

##### Case 2—Synopsis and Discussion

**Figure 3:**
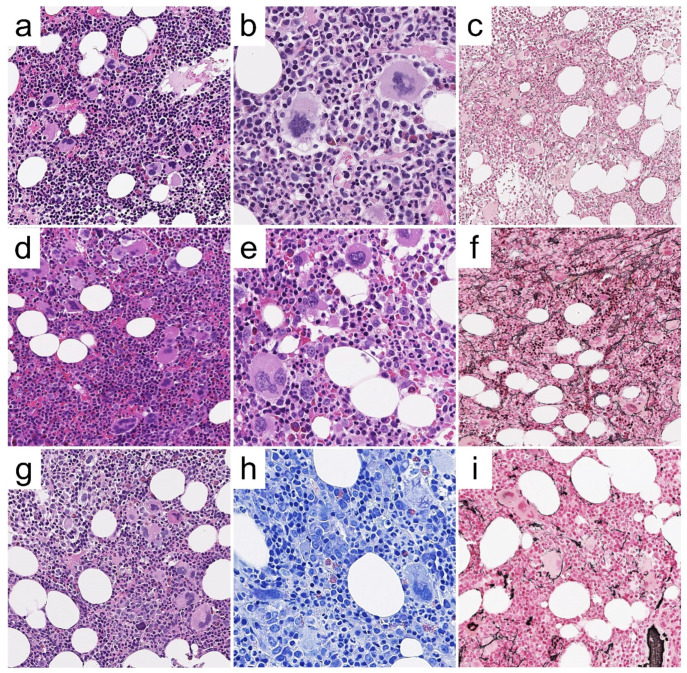
Representative histological features of case #2, #3 and #4. BM biopsy from case #2 (**a**–**c**) shows a hypercellular marrow with panmyelosis and loose clustering of megakaryocytes (**a**; H/E, 200×), featuring giant, hyperlobulated forms (**b**; H/E, 400×); reticulin network is unremarkable (**c**; Gomori silver stain, 200×). BM biopsy from case #3 (**d**–**f**) depicts hypercellularity with panmyelosis and loose to denser clustering of megakaryocytes (**d**; H/E, 200×), which range in morphology from small/hypolobulated to large/hyperlobulated (**e**, H/E, 400×); a minor increase in reticulin network, consistent with MF-1 grade is observed (**f**; Gomori silver stain, 200×). BM biopsy from case #4 (**g**–**i**) features panmyelosis and loose clustering of megakaryocytes with small to giant, hyperlobulated forms and naked nuclei (**g**; H/E, 200×); megaloblastoid features of erythropoiesis are best appreciated with Giemsa stain (**h**; 400×), whereas Gomori stain is unremarkable (**i**; 200×).

A 32-year old pregnant woman refers to the Emergency Unit for abdominal pain. Sonography reveals SVT of the splenic-portal axis and blood tests disclose anemia (Hb = 10.5 g/dL; Hct = 32%), leukocytosis (WBC = 18.2 × 10^9^/L), and thrombocytosis (Plt = 596 × 10^9^/L) with low Epo levels (3.3 mU/mL [4.3–29]), and *JAK2^V617F^* mutation. A BM biopsy (Figure 3a–c) discloses PV changes with hypercellularity, expansion of the three hematopoietic lineages, clustering of polymorphic megakaryocytes, and no fibrosis. Despite this, the lack of the first major criterion hampers a formal diagnosis of PV.

The present case depicts the prototypic features of “masked PV”, presenting with SVT. A further bias is the concurrent pregnancy, which itself favors deep vein thrombosis. Such a clinical event may occur in any subtype of MPN, either at diagnosis or during the course of the disease. A higher thrombotic risk is related to a hyperinflammatory state and neutrophilia, which are more commonly experienced in PV [43]. Besides the diagnostic issues, it should be stressed that thrombotic events pose the highest threat for patients with early phase MPN. Despite the risk factors for vascular events are only partially defined, major determinants include *JAK2* status, age, neutrophil count, and a positive history of thrombosis [43,44,45].

##### Case 3—Synopsis and Discussion

A 44-year old female with unremarkable past clinical history refers to the Hematology Clinic for persistent thrombocytosis (Hb = 15.2 g/dL; Hct = 44.8%; WBC = 12.65 × 10^9^/L [N = 9.58 × 10^9^/L]; Plt = 742 × 10^9^/L). Epo levels are close to the lower limit of normal (4.29 mU/mL; range: 4.3–29), LDH is not increased (206 U/L, range: 135–214) and *JAK2^V617F^* mutation is detected (VAF = 5.5%). Splenomegaly is not documented at clinical examination and sonography. BM biopsy (Figure 3d–f) discloses a hypercellular marrow with features of panmyelosis, maturing erythroid and myeloid lineages and polymorphous, and loosely clustering megakaryocytes with frequent giant hyperlobated forms. A minor increase in reticulin fibers is observed, consistent with MF-1 according to WHO criteria. This case displays borderline features between a pre-polycythemic phase of PV (highly supported by the very typical morphologic pattern) and pre-fibrotic PMF.

A diagnosis of ET is ruled out by BM histology. In contrast to case 1, the still low *JAK2^V617F^* allele burden could be responsible for the blood count, which is not fully consistent with PV. In similar cases, concurrent hematopoiesis-modifying factors (e.g., thalassemic trait, vitamin deficiencies, malabsorption, etc.) should be investigated. In addition, a minor increase in fibrosis should warrant closer monitoring.

##### Case 4—Synopsis and Discussion

A 79-year old woman with beta-thalassemia minor and IgG+ monoclonal gammopathy of undetermined potential presents with stable Hb levels and a steady increase of platelet count (Hb = 13.2 g/dL; Hct = 43.5%; WBC = 14.2 × 10^9^/L [N 9.67]; Plt = 866 × 10^9^/L; LDH levels within normal ranges, Epo not assessed). *JAK2^V617F^* mutation is detected on peripheral blood (VAF not known). A BM biopsy (Figure 3g–i) is hypercellular, with erythroid precursor expansion, megaloblastosis, and clustering of megakaryocytes, ranging in morphology from small, hypolobulated forms (including naked nuclei) to giant, hyperlobulated (“staghorn”) cells. No increase in reticulin fibers and CD34+ blasts is observed; the lympho-plasmacytic infiltrate is unremarkable.

The global picture strongly favors a diagnosis of MPN, but the differential diagnosis between ET and PV cannot be definitively settled. The former is favored by thrombocytosis and by prominent ET-type megakaryocyte morphology, and the latter is supported by the coexistence of neutrophilia alongside with normal Hb levels (possible effect of the concurrent thalassemic trait) [46].

#### 5.1.3. MPN with Unusual Morphology

In some cases, very unusual morphologic features or non-typical clinic-laboratory findings may challenge the diagnosis of MPN itself or suggest the differential diagnosis with other myeloid neoplasms (e.g., MDS/MPN).

##### Case 5—Synopsis and Discussion

**Figure 4:**
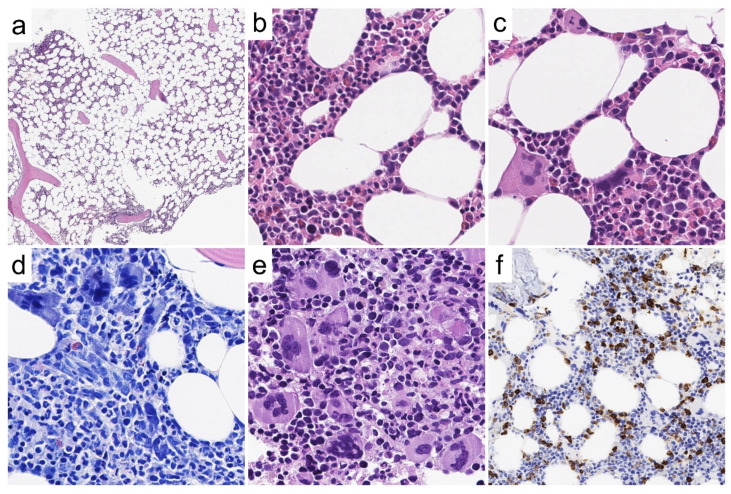
Representative histological features of case #5 and #6. BM biopsy from case #5 (**a**–**c**) shows a hypocellular marrow (**a**; H/E, 40×), featuring, at least focally, a mild decrease in myeloid-to-erythroid ratio without maturation defects (**b**; H/E, 400×), and scattered MPN-like, giant hyperlobulated megakaryocytes (**c**; H/E, 400×). BM biopsy from case #6 (**d**–**f**) depicts hypercellularity with increased myeloid-to-erythroid ratio, myeloid left shifting and clustering of megakaryocytes with hyperlobated forms and maturation defects (**d**; Giemsa, 400×; **e**; H/E 400×); a well-represented CD14+ monocyte population is present (**f**; 200×).

A 32-year old male with unremarkable past clinical history presents with persistent thrombocytosis (Hb = 15.6 g/dL; Hct = 46.1%; WBC = 6.76 × 10^9^/L; Plt = 745 × 10^9^/L; Epo and LDH levels within normal ranges). *JAK2^V617F^* mutation is detected (VAF = 10.1%) on peripheral blood granulocytes. A BM biopsy (Figure 4a–c) features striking hypocellularity without morphologic atypia. A slight decrease in the myeloid-to-erythroid ratio is noted with mildly increased, polymorphic megakaryocytes consisting of mature-looking to giant, hyperlobulated forms. There is no evidence of BM fibrosis and CD34+ blasts are not increased. The clinicopathologic synopsis supports MPN, but the very unusual morphologic picture favors a provisional diagnosis of MPN-U. Hypocellularity is uncommon in clonal myeloid disorders in general and barely reported in both early and chronic phase MPN. It may nonetheless be encountered in late-stage disease with the classical picture of MF with myeloid metaplasia. In this setting, the BM niche is overwhelmed by the stromal component with consequent peripheralization of hematopoietic cells. In the current case, the diagnosis of MPN is strongly supported by the laboratory findings, by the presence of a driver mutation, and by megakaryocyte morphology. Even though most clues point towards ET, the relative increase in the erythroid progenitors and Hb levels at the upper limit of normal cannot definitively rule out early phase PV with ET-like presentation and very atypical BM morphology.

##### Case 6—Synopsis and Discussion

An 80-year old male presents with longstanding monocytosis and progressive thrombocytosis without anemia (Hb = 13.4 g/dL; Hct = 41.3%; WBC 5.9 × 10^9^/L [monocytes 1.42 × 10^9^/L, 24%]; Plt = 1.348 × 10^9^/L). Epo levels are within normal limits, while LDH levels are increased (290 U/L; range: 135–225). Morphologic assessment of a BM smear is not informative due to absent cellularity, whereas a BM biopsy (Figure 4d–f) features hypercellularity with increased myeloid-to-erythroid ratio, myeloid left shifting, and a well-represented CD14+ monocyte component. The megakaryocytes display clustering with frequent giant hyperlobated forms. Small megakaryocytes with maturation defects are also present. Reticulin stain and blast count are unremarkable. Targeted next generation sequencing (NGS) on the peripheral blood cells documents mutations of *TET2* (VAF = 37%), *JAK2* (VAF = 26%) and *CALR* (VAF = 28%); the karyotype is normal.The clinic-pathologic picture is consistent with a myeloid neoplasm with monocytosis and poses the differential diagnosis between PMF and chronic myelomonocytic leukemia. While the overall histological picture is more in keeping with MPN, the left shifting of the myeloid series and the maturation defects of the megakaryocytes may, in contrast, suggest multi-lineage dysplasia. However, the mutational profile favors a JAK2/CALR-driven MPN. *TET2* mutations are of little value, as they can be present (likely as early clonal events) both in MPN, MDS/MPN, and in clonal hematopoiesis of indeterminate potential (CHIP) [47,48,49,50,51]. As to the unusual coexistence of driver gene mutations, a *JAK2/CALR* double-mutated genotype has been reported rarely in MPNs and does not correlate with specific clinical phenotypes [49,52]. Finally, it should be stressed that monocytosis in MPNs is mostly observed alongside fibrotic progression and may be assumed as an indicator of accelerated phase disease [53,54]. This was not the case for our patient.

### 5.2. MPN with Unconventional Molecular Features

Molecular genetics is a cardinal tool in the workup of MPNs, as it contributes to the diagnosis, classification, and prognostic stratification of these disorders. The identification of myeloid neoplasm-related gene derangements constitutes a major diagnostic criterion (i) to prove the clonal nature of the disease (particularly in TN cases); and/or (ii) to support/exclude specific disease subtypes [55]. With its increasing availability in the clinical setting, targeted NGS also allows the detection of atypical mutations in driver genes and of genetic derangements impacting on disease progression [56,57,58]. The quantitative assessment of VAF can also provide clinically relevant information on the disease burden and on the risk of adverse events. Indeed, the VAF of driver genes increases alongside myelofibrotic evolution, and high *JAK2^V617F^* burdens correlate with the risk of thrombosis [45,48,59,60]. Together with mutational analyses, karyotyping proves informative to assess the risk of progression, particularly in the myelofibrotic setting [61].

#### 5.2.1. *JAK2*-Negative Erythrocytosis

A definite diagnosis of PV should be rendered with great caution in the absence of identifiable *JAK2* mutations, as these occur in >95% of cases [1,62]. A common instance related to this scenario is the occurrence of cases with low-burden driver gene mutations (i.e., VAF falling below the sensitivity of the available techniques) [41]. Less frequently, a clinical and morphologic picture fully consistent with PV may be sustained by a *JAK2^wt^* clone. This imposes a challenging assessment of alternative congenital or acquired states of erythrocytosis [62]. Interestingly, sporadic *JAK2^wt^* MPN cases featuring the clinical phenotype of PV have been associated with mutations of *CALR*, either canonical (type 1) [63] or noncanonical [64]. Rare *JAK2^wt^* cases, some of which with PV features, may also carry mutations in *LNK/SH2B3* [65]. This genetic event has been associated with an increased risk of blastic transformation in MPN [66], as well as with occasional cases of idiopatic erythrocytosis [62].

#### 5.2.2. MPN with “High Risk” Molecular Features

While molecular testing is pivotal to guide MPN diagnosis [67], in rare instances it may yield puzzling results, which need to be interpreted after integration with clinical, laboratory, and histological data. This is specifically the case of genes rarely mutated in MPNs and associated with an aggressive clinical course.

##### Case 7—Synopsis and Discussion

**Figure 5:**
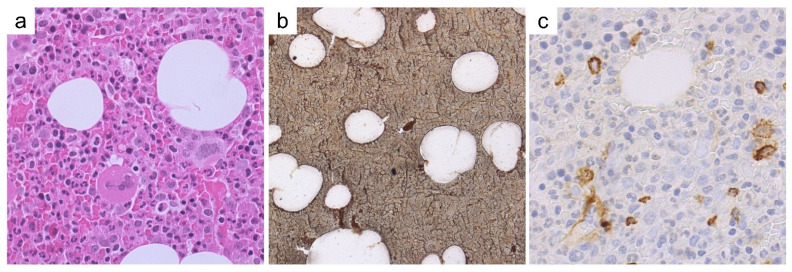
Representative histological features of case #7. BM biopsy from case #7 shows panmyelosis with normal myeloid-to-erythroid ratio, mildly left shifted granulopoiesis and increased, mature-looking megakaryocytes (**a**; H/E, 200×) with unremarkable reticulin network (**b**; Gomori silver stain, 200×) and a minor increase in CD34+ blasts (**c**; 200×).

A 46-year-old woman presents with a long-lasting history of thrombocytosis (mean platelets: 500 × 10^9^/L) and a putative clinical diagnosis of ET treated with low-dose aspirin. A BM biopsy (Figure 5) is performed 20 years after clinical onset and shows hypercellular, trilinear hematopoiesis with normal myeloid-to-erythroid ratio, mildly left shifted granulopoiesis, and increased, mature-looking megakaryocytes. Blasts account for 3–4% of the hematopoietic cells and reticulin fibrosis is not increased. Driver mutations of *JAK2*, *CALR* and *MPL* are not detected, but targeted NGS documents the L56S *RUNX1* variant. The overall morphological and molecular findings exclude ET and suggest an alternative diagnosis of *RUNX1*-mutated myeloid neoplasm with thrombocytosis.

Germline *RUNX1* mutations are reported in inherited forms of platelet disorders with a predisposition to myeloid malignancies [68]. Acquired *RUNX1* derangements are instead relatively common in MDS, MDS/MPN, AML, and in contexts akin to CHIP [69]. In the present case, the unremarkable family history, the clinical presentation, and the histological findings exclude such diagnoses and favors MPN-U. Of note, rare cases of MPN feature *RUNX1* mutations and typically undergo myelofibrotic and/or blastic progression [58,70]. This and 3 similar cases published by Cattaneo and colleagues [55] disclose the unique features of an MPN with “high risk” genetics and an indolent, ET-like clinical course.

#### 5.2.3. MPN with CHIP-like Molecular Features

A share of TN MPNs discloses variants of myeloid neoplasm-/CHIP-associated genes (i.e., *DNMT3A*, *TET2*, and *ASXL1*) with ascertained or putative pathogenicity [58,71,72].

From a clinical perspective, an overlap between CHIP and early TN MPN is sustained by the indolent clinical course and by the slightly increased thrombotic risk of both conditions [48,73,74]. In line with this, by applying highly sensitive molecular techniques, we could document CHIP-related changes in >80% of TN MPNs with ET-like presentation and chronic-indolent follow up [55]. This data points toward a close relationship between CHIP and some early phase TN MPNs. They also pose intriguing questions on the boundaries between such entities.

It should also be noted that large cohort studies on TN MPNs [56] and CHIP [75,76] report high allele burdens for the above-mentioned variants in a substantial fraction of patients, implying a relevant role for such (even “high risk”) imbalances in sustaining clonal hematopoiesis.

Overall, subsets of CHIP may thus represent precursor hematopoietic clones, characterized by normal blood cell counts and intrinsic potential to TN MPN evolution [55,72]. Further clinical-pathological and molecular studies are needed to test this fascinating possibility.

## 6. Conclusions

Philadelphia-negative MPNs are a heterogeneous group of hematopoietic disorders, characterized by distinct clinical and biological features. Over the years, the classification and diagnostic criteria of such entities have undergone major changes and have been progressively refined. Despite this, a small (yet consistent) subset of cases lacks a precise definition. Such MPN-Us represent either early or advanced disease stages, as well as MPNs with hardly classifiable biological features. A precise characterization of these entities is made possible by the thorough integration of clinical, histological, and molecular studies. This approach will hopefully contribute to a better understanding of MPN biology and to further improvements in the clinical managements of patients.

## Figures and Tables

**Table 2:** Evolution of diagnostic criteria for Polycythemia Vera.

*PVSG Criteria*	*WHO Criteria 2001*	*WHO Criteria 2008*	*WHO Criteria 2016*
***Major criteria*** A1. Increased red cell mass (RCM; M ≥ 36 mL/Kg; F ≥ 32 mL/Kg) A2. Oxygen saturation ≥92% A3. Splenomegaly ***Minor criteria*** B1. Platelets ≥400 × 10^9^/L B2. WBC ≥12 × 10^9^/L B3. LAP score >100 B4. Serum vitamin B_12_ >900 pg/mL; unbound vitamin B_12_ >2200 pg/mL	***Major criteria*** A1. Increased RCM (>25% of expected) or Hb levels (M > 18.5 g/dL; F > 16.5 g/dL) A2. No causes of secondary erythrocytosis A3. Splenomegaly A4. Clonal genetic abnormality other than BCR/ABL1 fusion or Philadelphia chromosome A5. Erythroid colony formation in vitro ***Minor criteria*** B1. Platelets ≥400 × 10^9^/L B2. WBC ≥12 × 10^9^/L B3. Consistent BM findings B4. Low serum EPO levels	***Major criteria*** Increased Hb (M > 18.5 g/dL; F > 16.5 g/dL) * V617F or similar JAK2 mutations ***Minor criteria*** Consistent BM findings Low serum EPO levels Erythroid colony formation in vitro	***Major criteria*** Increased Hb (M > 16.5 g/dL; F > 16.0 g/dL) or Htc (M > 49%; F > 48%) or RCM (>25% of predicted values) Consistent BM findings V617F or exon 12 JAK2 mutations ***Minor criterion*** Low serum EPO levels
Diagnosis posed if A1 + A2 + A3 or A1 + A2 and 2 B criteria are fulfilled	Diagnosis posed if A1 + A2 and any other A or A1 + A2 and 2 B criteria are fulfilled	Diagnosis posed if both the major and one minor criteria or the first major and 2 minor criteria are fulfilled	Diagnosis posed if all major criteria or the first 2 major and the minor criteria are fulfilled

Notes: (*) additional criteria for increased erythroid burden include Hb or Htc >99% percentile and Hb >17 g/dL in M or 15 g/dL in F if associated with a sustained increase ≥2 g/dL.

**Table 3:** Evolution of diagnostic criteria for Essential Thrombocythemia.

*PVSG Criteria*	*WHO Criteria 2001*	*WHO Criteria 2008*	*WHO Criteria 2016*
A1. Platelets ≥600 × 10^9^/L A2. No known cause of reactive thrombocytosis A3. Normal Hb and RCM A4. No BM features of MDS A5. Absence of BCL/ABL1 fusion or Philadelphia chromosome A6. Collagen fibrosis absent or in <1/3 of BM area w/o marked splenomegaly and leukoerythroblastosis	***Positive criteria*** Platelets ≥600 × 10^9^/L Consistent BM findings ***Criteria of exclusion*** No evidence of PV No evidence of CML No evidence of PMF No evidence of MDS No evidence of reactive thrombocytosis due to inflammation, infection, neoplasm or prior splenectomy	Platelets >450 × 10^9^/L Consistent BM findings Not meeting WHO for CML, PV, PMF, MDS or other myeloid neoplasms V617F JAK2 mutation or other clonal markers or no evidence of reactive thrombocytosis	***Major criteria*** Platelets ≥450 × 10^9^/L Consistent BM findings Not meeting WHO for CML, PV, PMF, MDS or other myeloid neoplasms JAK2 CALR or MPL mutations ***Minor criterion*** Presence of a clonal marker or no evidence of reactive thrombocytosis
Diagnosis posed if all criteria are fulfilled	Diagnosis posed if all criteria are fulfilled	Diagnosis posed if all criteria are fulfilled	Diagnosis posed if all major criteria or the first 3 major and the minor criteria are fulfilled

**Table 4:** Evolution of diagnostic criteria for Primary Myelofibrosis.

WHO Criteria 2001	WHO Criteria 2008	WHO Criteria 2016
**Prefibrotic phase**	**Prefibrotic phase**	**Prefibrotic phase**
***Clinical findings*** No or mild splenomegaly/hepatomegaly Mild anemia Variable leukocyte and platelet counts ***Morphological findings in PB*** No or mild leukoerythroblastosis No or little poikilocytosis and dacrocytosis ***Morphological findings in BM*** Increased cellularity Increased atypical megakaryocytes Neutrophilic proliferation Minimal to absent reticulin firbosis	***Major criteria*** Increased BM cellularity with increased atypical megakaryocytes and expanded granulopoiesis No evidence of PV, CML, MDS or other myeloid neoplasms 3. JAK2 V617F mutation or other clonal markers; if clonality not detected, all causes of secondary fibrosis must be excluded ***Minor criteria*** Leukoerythroblastosis Increased LDH levels Anemia Splenomegaly	***Major criteria*** Increased atypical megakaryocytes, fibrosis grade ≤1, increased cellularity and granulopoiesis No evidence of PV, ET, CML, MDS or other myeloid neoplasms 3. JAK2, CALR or MPL mutations or other clonal markers or no evidence of reactive BM fibrosis ***Minor criteria*** WBC ≥11 × 10^9^/L Increased LDH levels Anemia Splenomegaly
**Fibrotic phase**	**Fibrotic phase**	**Fibrotic phase**
***Clinical findings*** Moderate to marked splenomegaly Moderate to marked hepatomegaly Moderate to marked anemia Variable leukocyte and platelet counts ***Morphological findings in PB*** Leukoerythroblastosis Poikilocytosis and dacrocytosis ***Morphological findings in BM*** Reticulin or collagen fibrosis; Reduced cellularity Dilated sinusoids Increased atypical megakaryocytes Osteosclerosis	***Major criteria*** Increased atypical megakaryocytes with reticulin and/or collagen fibrosis No evidence of PV, CML, MDS or other myeloid neoplasms JAK2 V617F mutation or other clonal markers; if clonality not detected, all causes of secondary fibrosis must be excluded ***Minor criteria*** Leukoerythroblastosis Increased LDH levels Anemia Splenomegaly	***Major criteria*** Increased atypical megakaryocytes, fibrosis grade >1 No evidence of PV, ET, CML, MDS or other myeloid neoplasms JAK2, CALR or MPL mutations or other clonal markers or no evidence of reactive BM fibrosis ***Minor criteria*** WBC ≥11 × 10^9^/L Increased LDH levels Anemia Splenomegaly Leukoerythroblastosis
Minimal criteria for diagnosis not formally provided	Diagnosis posed if all major and 2 minor criteria are fulfilled	Diagnosis posed if all major and ≥1 minor criterion are fulfilled

## References

[1] Swerdlow S.H., Campo E., Harris N.L., Jaffe E.S., Pileri S.A., Stein H., Thiele J. (2017). WHO Classification of Tumours of Haematopoietic and Lymphoid Tissues.

[2] Anderson L.A., McMullin M.F. (2014). Epidemiology of MPN: What Do We Know?. Curr. Hematol. Malign. Rep..

[3] Szuber N., Mudireddy M., Nicolosi M., Penna D., Vallapureddy R.R., Lasho T.L., Finke C., Begna K.H., Elliott M.A., Hook C.C. (2019). 3023 Mayo Clinic Patients with Myeloproliferative Neoplasms: Risk-Stratified Comparison of Survival and Outcomes Data Among Disease Subgroups. Mayo Clin. Proc..

[4] Tefferi A. (2007). The history of myeloproliferative disorders: Before and after Dameshek. Leukemia.

[5] Dameshek W. (1951). Some speculations on the myeloproliferative syndromes. Blood.

[6] Ward H.P., Block M.H. (1971). The natural history of agnogenic myeloid metaplasia (AMM) and a critical evaluation of its relationship with the myeloproliferative syndrome. Medicine.

[7] Arber D.A. (2019). The 2016 WHO classification of acute myeloid leukemia: What the practicing clinician needs to know. Semin. Hematol..

[8] Mughal T.I., Abdel-Wahab O., Rampal R., Mesa R., Koschmieder S., Levine R., Hehlmann R., Saglio G., Barbui T., Van Etten R.A. (2016). Contemporary insights into the pathogenesis and treatment of chronic myeloproliferative neoplasms. Leuk. Lymphoma.

[9] Mathé G., Rappaport H. (1976). Histological and Cytological Typing of Neoplastic Diseases of Haematopoietic and Lymphoid Tissues.

[10] Burkhardt R., Bartl R., Jäger K., Frisch B., Kettner G., Mahl G., Sund M. (1984). Chronic Myeloproliferative Disorders (CMPD). Pathol. Res. Pr..

[11] Georgii A., Vykoupil K.-F., Buhr T., Choritz H., Döhler U., Kaloutsi V., Werner M. (1990). Chronic Myeloproliferative Disorders in Bone Marrow Biopsies. Pathol. Res. Pr..

[12] Thiele J., Kvasnicka H.-M., Werden C., Zankovich R., Diehl V., Fischer R. (1996). Idiopathic Primary Osteo-myelofibrosis: A Clinico-Pathological Study on 208 Patients with Special Emphasis on Evolution of Disease Features, Differentiation from Essential Thrombocythemia and Variables of Prognostic Impact. Leuk. Lymphoma.

[13] Szuber N., Tefferi A. (2018). Chronic neutrophilic leukemia: New science and new diagnostic criteria. Blood Cancer J..

[14] Cassi E., De Paoli A., Fava S., Luoni M., Tosi A., Turri C., Grimi E. (1992). Idiopathic hypereosinophilic syndrome and “eosinophilic leukemia”. Haematologica.

[15] Jaffe E.S., Harris N.L., Stein H., Vardiman J.W. (2001). Pathology and Genetics of Tumours of Haematopoietic and Lymphoid Tissues.

[16] Harris N.L., Jaffe E.S., Stein H., Banks P.M., Chan J.K., Cleary M.L., Delsol G., De Wolf-Peeters C., Falini B., Gatter K.C. (1994). A revised European-American classification of lymphoid neoplasms: A proposal from the International Lymphoma Study Group. Blood.

[17] Spivak J.L., Silver R.T. (2008). The revised World Health Organization diagnostic criteria for polycythemia vera, essential thrombocytosis, and primary myelofibrosis: An alternative proposal. Blood.

[18] Vainchenker W., Kralovics R. (2017). Genetic basis and molecular pathophysiology of classical myeloproliferative neoplasms. Blood.

[19] Longley B.J., Tyrrell L., Lu S.-Z., Ma Y.-S., Langley K., Ding T.-G., Duffy T., Jacobs P., Tang L.H., Modlin I. (1996). Somatic c-KIT activating mutation in urticaria pigmentosa and aggressive mastocytosis: Establishment of clonality in a human mast cell neoplasm. Nat. Genet..

[20] Reiter A., Gotlib J. (2017). Myeloid neoplasms with eosinophilia. Blood.

[21] Swerdlow S.H., Campo E., Harris N.L., Jaffe E.S., Pileri S., Stein H., Thiele J., Vardiman J.W. (2008). WHO Classification of Tumours of Haematopoietic and Lymphoid Tissues.

[22] Tefferi A., Thiele J., Orazi A., Kvasnicka H.M., Barbui T., Hanson C.A., Barosi G., Verstovsek S., Birgegard G., Mesa R. (2007). Proposals and rationale for revision of the World Health Organization diagnostic criteria for polycythemia vera, essential thrombocythemia, and primary myelofibrosis: Recommendations from an ad hoc international expert panel. Blood.

[23] Arber D.A., Orazi A., Hasserjian R., Thiele J., Borowitz M.J., Le Beau M.M., Bloomfield C.D., Cazzola M., Vardiman J.W. (2016). The 2016 revision to the World Health Organization classification of myeloid neoplasms and acute leukemia. Blood.

[24] Wasserman L.R. (1971). THE MANAGEMENT OF POLYCYTHAEMIA VERA. Br. J. Haematol..

[25] Murphy S., Iland H., Rosenthal D., Laszlo J. (1986). Essential thrombocythemia: An interim report from the Polycythemia Vera Study Group. Semin. Hematol..

[26] Michiels J. (1997). Diagnostic criteria of the myeloproliferative disorders (MPD): Essential thrombocythaemia, polycythaemia vera and chronic megakaryocytic granulocytic metaplasia. Neth. J. Med..

[27] Barbui T., Thiele J., Carobbio A., Guglielmelli P., Rambaldi A., Vannucchi A.M., Tefferi A. (2014). Discriminating between essential thrombocythemia and masked polycythemia vera inJAK2mutated patients. Am. J. Hematol..

[28] Barbui T., Thiele J., Gisslinger H., Kvasnicka H.M., Vannucchi A.M., Guglielmelli P., Orazi A., Tefferi A. (2018). The 2016 WHO classification and diagnostic criteria for myeloproliferative neoplasms: Document summary and in-depth discussion. Blood Cancer J..

[29] Gisslinger B., Jeryczynski G., Wolfler A., Burgstaller S., Buxhoferausch V., Schalling M., Krauth M.-T., Schiefer A.-I., Kornauth C., Simonitschklupp I. (2015). Clinical impact of bone marrow morphology for the diagnosis of essential thrombocythemia: Comparison between the BCSH and the WHO criteria. Leukemia.

[30] Thiele J., Kvasnicka H.M. (2003). Chronic myeloproliferative disorders with thrombocythemia: A comparative study of two classification systems (PVSG, WHO) on 839 patients. Ann. Hematol..

[31] Florena A.M., Tripodo C., Iannitto E., Porcasi R., Ingrao S., Franco V. (2004). Value of bone marrow biopsy in the diagnosis of essential thrombocythemia. Haematologica.

[32] Thiele J., Kvasnicka H.M., Diehl V., Fischer R., Michiels J.J. (1999). Clinicopathological Diagnosis and Differential Criteria of Thrombocythemias in Various Myeloproliferative Disorders by Histopathology, Histochemistry and Immunostaining from Bone Marrow Biopsies. Leuk. Lymphoma.

[33] Gianelli U., Cattaneo D., Bossi A., Cortinovis I., Boiocchi L., Liu Y.-C., Augello C., Bonometti A., Fiori S., Orofino N. (2017). Erratum: The myeloproliferative neoplasms, unclassifiable: Clinical and pathological considerations. Mod. Pathol..

[34] Primignani M., Barosi G., Bergamaschi G., Gianelli U., Fabris F., Reati R., Dell’Era A., Bucciarelli P., Mannucci P.M. (2006). Role of theJAK2 mutation in the diagnosis of chronic myeloproliferative disorders in splanchnic vein thrombosis. Hepatology.

[35] Iurlo A., Gianelli U., Cattaneo D., Thiele J., Orazi A. (2017). Impact of the 2016 revised WHO criteria for myeloproliferative neoplasms, unclassifiable: Comparison with the 2008 version. Am. J. Hematol..

[36] Gianelli U., Iurlo A., Cattaneo D., Bossi A., Cortinovis I., Augello C., Moro A., Savi F., Castelli R., Brambilla C. (2015). Discrepancies between bone marrow histopathology and clinical phenotype in BCR-ABL1-negative myeloproliferative neoplasms associated with splanchnic vein thrombosis. Leuk. Res..

[37] Deschamps P., Moonim M., Radia D., Curto-Garcia N., Woodley C., Bassiony S., O’Sullivan J., Harrington P., Raj K., Francis Y. (2021). Clinicopathological characterisation of myeloproliferative neoplasm-unclassifiable (MPN-U): A retrospective analysis from a large UK tertiary referral centre. Br. J. Haematol..

[38] Gianelli U., Bossi A., Cortinovis I., Sabattini E., Tripodo C., Boveri E., Moro A., Valli R., Ponzoni M., Florena A.M. (2013). Reproducibility of the WHO histological criteria for the diagnosis of Philadelphia chromosome-negative myeloproliferative neoplasms. Mod. Pathol..

[39] Kvasnicka H.M., Orazi A., Thiele J., Barosi G., Bueso-Ramos C.E., Vannucchi A.M., Hasserjian R.P., Kiladjian J.-J., Gianelli U., Silver R. (2017). European LeukemiaNet study on the reproducibility of bone marrow features in masked polycythemia vera and differentiation from essential thrombocythemia. Am. J. Hematol..

[40] Thiele J., Orazi A., Kvasnicka H.M., Franco V., Boveri E., Gianelli U., Gisslinger H., Passamonti F., Tefferi A., Barbui T. (2012). European Bone Marrow Working Group trial on reproducibility of World Health Organization criteria to discriminate essential thrombocythemia from prefibrotic primary myelofibrosis. Haematologica.

[41] Perricone M., Polverelli N., Martinelli G., Catani L., Ottaviani E., Zuffa E., Franchini E., Dizdari A., Forte D., Sabattini E. (2017). The relevance of a low JAK2V617F allele burden in clinical practice: A monocentric study. Oncotarget.

[42] Jovanovic J.V., Ivey A., Vannucchi A.M., Lippert E., Leibundgut E.O., Cassinat B., Pallisgaard N., Maroc N., Hermouet S., Nickless G. (2013). Establishing optimal quantitative-polymerase chain reaction assays for routine diagnosis and tracking of minimal residual disease in JAK2-V617F-associated myeloproliferative neoplasms: A joint European LeukemiaNet/MPN&MPNr-EuroNet (COST action BM0902) study. Leukemia.

[43] Carobbio A., Ferrari A., Masciulli A., Ghirardi A., Barosi G., Barbui T. (2019). Leukocytosis and thrombosis in essential thrombocythemia and polycythemia vera: A systematic review and meta-analysis. Blood Adv..

[44] Tefferi A., Barbui T. (2020). Polycythemia vera and essential thrombocythemia: 2021 update on diagnosis, risk-stratification and management. Am. J. Hematol..

[45] Debureaux P.-E., Cassinat B., Soret-Dulphy J., Mora B., Verger E., Maslah N., Plessier A., Rautou P.-E., Ollivier-Hourman I., De Ledinghen V. (2020). Molecular profiling and risk classification of patients with myeloproliferative neoplasms and splanchnic vein thromboses. Blood Adv..

[46] Kottas K., Marathonitis A., Nodarou A., Kanellis G., Christopoulos K. (2020). Polycythemia Vera in a Patient With Heterozygous Beta-Thalassemia: Coincidence or Causal Relationship?. Cureus.

[47] Delhommeau F., Dupont S., Della Valle V., James C., Trannoy S., Massé A., Kosmider O., Le Couedic J.-P., Robert F., Alberdi A. (2009). Mutation inTET2in Myeloid Cancers. N. Engl. J. Med..

[48] Steensma D.P., Bejar R., Jaiswal S., Lindsley R.C., Sekeres M., Hasserjian R.P., Ebert B.L. (2015). Clonal hematopoiesis of indeterminate potential and its distinction from myelodysplastic syndromes. Blood.

[49] Kang M.-G., Choi H.-W., Lee J.H., Choi Y.J., Shin J.-H., Suh S.-P., Szardenings M., Kim H.-R., Shin M.-G., Choi H.-J. (2016). Coexistence of JAK2 and CALR mutations and their clinical implications in patients with essential thrombocythemia. Oncotarget.

[50] Palomo L., Meggendorfer M., Hutter S., Twardziok S., Ademà V., Fuhrmann I., Fuster-Tormo F., Xicoy B., Zamora L., Acha P. (2020). Molecular landscape and clonal architecture of adult myelodysplastic/myeloproliferative neoplasms. Blood.

[51] Chia Y., Islam A., Hider P., Woon P., Johan M., Hassan R., Ramli M. (2021). The Prevalence of *TET2* Gene Mutations in Patients with *BCR*-*ABL*-Negative Myeloproliferative Neoplasms (MPN): A Systematic Review and Meta-Analysis. Cancers.

[52] Ahmed R.Z., Rashid M., Ahmed N., Nadeem M., Shamsi T.S. (2016). Coexisting JAK2V617F and CALR Exon 9 Mutations in Myeloproliferative Neoplasms—Do They Designate a New Subtype?. Asian Pac. J. Cancer Prev..

[53] Boiocchi L., Espinal-Witter R., Geyer J.T., Steinhilber J., Bonzheim I., Knowles D.M., Fend F., Orazi A. (2012). Development of monocytosis in patients with primary myelofibrosis indicates an accelerated phase of the disease. Mod. Pathol..

[54] Mangaonkar A.A., Tande A.J., Bekele D.I. (2021). Differential Diagnosis and Workup of Monocytosis: A Systematic Approach to a Common Hematologic Finding. Curr. Hematol. Malign- Rep..

[55] Cattaneo D., Croci G.A., Bucelli C., Tabano S., Cannone M.G., Gaudioso G., Barbanti M.C., Barbullushi K., Bianchi P., Fermo E. (2021). Triple-Negative Essential Thrombocythemia: Clinical-Pathological and Molecular Features. A Single-Center Cohort Study. Front. Oncol..

[56] Acha P., Xandri M., Fuster-Tormo F., Palomo L., Xicoy B., Cabezón M., Marcé S., Granada I., Vela D., Sagüés M. (2019). Diagnostic and prognostic contribution of targeted NGS in patients with triple-negative myeloproliferative neoplasms. Am. J. Hematol..

[57] Feenstra J.D.M., Nivarthi H., Gisslinger H., Leroy E., Rumi E., Chachoua I., Bagienski K., Kubesova B., Pietra D., Gisslinger B. (2016). Whole-exome sequencing identifies novel MPL and JAK2 mutations in triple-negative myeloproliferative neoplasms. Blood.

[58] Grinfeld J., Nangalia J., Baxter E.J., Wedge D., Angelopoulos N., Cantrill R., Godfrey A.L., Papaemmanuil E., Gundem G., MacLean C. (2018). Classification and Personalized Prognosis in Myeloproliferative Neoplasms. N. Engl. J. Med..

[59] Cazzola M., Kralovics R. (2014). From Janus kinase 2 to calreticulin: The clinically relevant genomic landscape of myeloproliferative neoplasms. Blood.

[60] Loscocco G., Coltro G., Guglielmelli P., Vannucchi A. (2021). Integration of Molecular Information in Risk Assessment of Patients with Myeloproliferative Neoplasms. Cells.

[61] Iurlo A., Palandri F., Elli E.M., Cattaneo D., Bucelli C., Sciumè M., Vincelli D., Brioschi F., Auteri G., Croci G.A. (2020). Cytogenetic study in primary myelofibrosis at diagnosis: Clinical and histological association and impact on survival according to WHO 2017 classification in an Italian multicenter series. Hematol. Oncol..

[62] Gangat N., Szuber N., Pardanani A., Tefferi A. (2021). JAK2 unmutated erythrocytosis: Current diagnostic approach and therapeutic views. Leukemia.

[63] Broséus J., Park J.-H., Carillo S., Hermouet S., Girodon F. (2014). Presence of calreticulin mutations in JAK2-negative polycythemia vera. Blood.

[64] Quattrocchi A., Maiorca C., Billi M., Tomassini S., De Marinis E., Cenfra N., Equitani F., Gentile M., Ceccherelli A., Banella C. (2020). Genetic lesions disrupting calreticulin 3′-untranslated region in JAK2 mutation-negative polycythemia vera. Am. J. Hematol..

[65] Maslah N., Cassinat B., Verger E., Kiladjian J.-J., Velazquez L. (2017). The role of LNK/SH2B3 genetic alterations in myeloproliferative neoplasms and other hematological disorders. Leukemia.

[66] Pardanani A., Lasho T.L., Finke C.M., Oh S.T., Gotlib J., Tefferi A. (2010). LNK mutation studies in blast-phase myeloproliferative neoplasms, and in chronic-phase disease with TET2, IDH, JAK2 or MPL mutations. Leukemia.

[67] Skov V. (2020). Next Generation Sequencing in MPNs. Lessons from the Past and Prospects for Use as Predictors of Prognosis and Treatment Responses. Cancers.

[68] Brown A.L., Arts P., Carmichael C., Babic M., Dobbins J., Chong C.-E., Schreiber A.W., Feng J., Phillips K., Wang P.P.S. (2020). RUNX1-mutated families show phenotype heterogeneity and a somatic mutation profile unique to germline predisposed AML. Blood Adv..

[69] Young A.L., Challen G.A., Birmann B., Druley T.E. (2016). Clonal haematopoiesis harbouring AML-associated mutations is ubiquitous in healthy adults. Nat. Commun..

[70] Bejar R., Stevenson K., Abdel-Wahab O., Galili N., Nilsson B., Garcia-Manero G., Kantarjian H., Raza A., Levine R.L., Neuberg D. (2011). Clinical Effect of Point Mutations in Myelodysplastic Syndromes. N. Engl. J. Med..

[71] Schischlik F., Jäger R., Rosebrock F., Hug E., Schuster M., Holly R., Fuchs E., Feenstra J.D.M., Bogner E., Gisslinger B. (2019). Mutational landscape of the transcriptome offers putative targets for immunotherapy of myeloproliferative neoplasms. Blood.

[72] Kjær L. (2020). Clonal Hematopoiesis and Mutations of Myeloproliferative Neoplasms. Cancers.

[73] Jaiswal S., Fontanillas P., Flannick J., Manning A., Grauman P.V., Mar B., Lindsley C., Mermel C., Burtt N., Chavez A. (2014). Age-Related Clonal Hematopoiesis Associated with Adverse Outcomes. N. Engl. J. Med..

[74] Veninga A., De Simone I., Heemskerk J.W., Cate H.T., Van Der Meijden P.E. (2020). Clonal hematopoietic mutations linked to platelet traits and the risk of thrombosis or bleeding. Haematologica.

[75] Buscarlet M., Provost S., Zada Y.F., Barhdadi A., Bourgoin V., Lépine G., Mollica L., Szuber N., Dubé M.-P., Busque L. (2017). DNMT3A and TET2 dominate clonal hematopoiesis and demonstrate benign phenotypes and different genetic predispositions. Blood.

[76] Zink F., Stacey S.N., Norddahl G.L., Frigge M.L., Magnusson O.T., Jonsdottir I., Thorgeirsson T.E., Sigurdsson A., Gudjonsson S.A., Gudmundsson J. (2017). Clonal hematopoiesis, with and without candidate driver mutations, is common in the elderly. Blood.

